# Observations on the Medical Use and Effect of the Digitalis Purpurea; Extracted from an Essay of Dr. Mease, Resident Physician of the Port of Philadelphia

**Published:** 1799-03

**Authors:** 


### *Obfervations on the Medical Ufe and Effect of the Digitalis Purpurea; extracted from an Effay of* Dr. MEASE, *Refident Phyfician of the Port of Philadelphia.*

THE Purple Fox-glove is a medicine which, for fome time, ftood high in the lift of the materia medica, but for various reafons appears to have, in fome meafure, loft the character once formed of it.—The caufes influencing the fuccefs of the *digitalis*, may be referred to the following heads:

heads: 1. The *ſeaſon of the year in which the plant is collected* will have a very conſiderable influence on the effects produced on the ſyſtem. 2. The *part of the plant employed* ought likewiſe to be attended to, when forming a judgment of its virtues. 3. A frequent cauſe of the failure of *digitalis* may be attributed to the careleſs mode of preparing it for uſe. 4. The *adulteration of the plant* with other vegetables. 5. The various mode of exhibiting this medicine; and 6. the *particular condition* of *the patient* at the *time* of its *exhibition.*

Dr. Mease very judiciouſly comments upon each of theſe particulars, and more at large upon the laſt. He examines with ſome acuteneſs the different opinions of phyſicians, relative to this ſubject, and illuſtrates them with his own obſervation and experience on the effects of this powerful plant.—As a ſpecimen of his method of reaſoning, we ſhall quote the following remarkable paſſage:

"To account for the ſucceſs of this remedy in dropſies, and diſeaſes of too much action in the arterial ſyſtem, is readily done. But, to reconcile the oppoſite teſtimony of Dr. Withering and others, with reſpect to their different ſucceſs in dropſies of too little action, is very difficult: nay, to reconcile the contrary advice of Dr. Withering himſelf, who recommends the digitalis in mania and hemoptœ, 'with a *bounding pulſe*', and in dropſies with a *weak* one, is impoſſible. And here I may repeat a remark I formerly made, viz. 'That a medicine whoſe primary operation is the reduction of the force of the arterial ſyſtem, when this is ſo intimately connected with the general ſtrength of the body, ſhould only ſucceed in thoſe caſes, where the latter is conſiderably exhauſted, appears not only to be paradoxical, but ill agrees with the uniformity obſerved in the operations of nature, in other parts of the animal œconomy.'—It is incumbent on Dr. Withering to account for this apparent oppoſition in his own ſentiments, and to ſatisfy the doubts of phyſicians, ariſing from the action of the remedy being directly contrary to the principles upon which alone certain dropſies can be cured. I may alſo remark that it is much to be wiſhed, phyſicians were more accurate in noting the ſtate of their patients ſyſtem, at the time of preſcribing this and other remedies; and alſo the ſtate of the pulſe at the ſame time, with its frequency; which, though ſo conſtantly ſtated, is of far leſs conſequence in determining the ſituation of the ſyſtem, as to the important point of the exiſtence or abſence of the inflammatory diſpoſition, than the former. We ſhould thus arrive with more certainty at the *true* virtues of a medicine, and be able to reconcile the contradictory accounts of phyſicians, reſpecting its utility in the ſame diſeaſe."

Theſe

Thefe *golden* remarks cannot be too *often* and too *ferioufly* repeated, particularly to the junior clafs of medical practitioners.—Our author quotes a ftriking paffage from a letter of Dr. Withering to Dr. Woodville, publifhed in the "*Medical Botany*" of the latter, in which the inattention of phyficians, in the exhibition of *digitalis* is feverely cenfured, and he concludes with the following practical hints:

"It may not be improper to add fome cautions and obfervations upon the ufe of digitalis, not mentioned in the above paper, for the information of thofe who may not have feen Dr. Withering's work, and may wifh to exhibit the medicine.

"The Doctor defires the medicine to be given in the dofes in an infufion of the plant, which he prefers to every other form, and directs one drachm of the dried leaves to be infufed for four hours in half a pint of boiling water, adding to the ftrained liquor half an ounce of any fpirituous water, and an ounce of this infufion to be given twice a day to an adult. If the patient be ftronger than ufual, or the fymptoms very urgent, this dofe may be given once in eight hours; and, on the contrary, in many inftances, half an ounce at a time will be quite fufficient. (This method of exhibiting the medicine, ought to be continued, until it either acts on the kidneys or ftomach, pulfe or bowels: to be ftopped upon the firft appearance of any of thefe effects; the patient to drink freely during its operation.) But, without attending to this rule, I have known the digitalis prefcribed in the various forms of decoction, pills made with foap, and in powder mixed with prepared chalk or magnefia; and though the two laft fubftances may not act in any oppofite way, yet they may divide the particles, and, by clogging them, prevent application to the ftomach, and thus diminifh its activity. A tedious decoction will diffipate the virtue of many plants lefs volatile than digitalis; but, a flight one will no doubt injure it, and ought therefore never to be employed. Pills are often made up with fuch fubftances, as render them foluble with difficulty. If we even fuppofe foap free from this objection, we neverthelefs do not know, what fecret effects may be produced by it, on particular ftomachs, to interrupt the action of the medicine; and certainly it will not be fo readily applied to that organ, as in a watery form.

"In cafe of *afcites* and *anafarca*, when the patient is weak, and the evacuation of the water rapid, the ufe of a proper bandage is indifpenfably neceffary. If the water fhould not be wholly evacuated, it is beft to allow an interval of feveral days, before the medicine be repeated, in order that food and tonics may be exhibited.

"The

"The flow of urine will often precede, ſometimes accompany, and frequently follow the ſickneſs at ſtomach, at the diſtance of ſome days, and not unfrequently be checked by the medicine, eſpecially if given in too large doſes. The ſickneſs will ſometimes ceaſe, and recur again as violent as before, and will continue to recur for three or four days, at more diſtant intervals. Theſe ſufferings ſeldom occur, and are no objection to the uſe of digitalis, neither is a ſtone in the bladder."

